# Targeting cell death pathways in intestinal ischemia-reperfusion injury: a comprehensive review

**DOI:** 10.1038/s41420-024-01891-x

**Published:** 2024-03-04

**Authors:** Fei Wang, Huiming Huang, Xuejiao Wei, Peng Tan, Zhuguo Wang, Zhongdong Hu

**Affiliations:** 1 School of Chinese Materia Medica, Beijing University of Chinese Medicine, 100029 Beijing, China; 2 Modern Research Center for Traditional Chinese Medicine, Beijing Research Institute of Chinese Medicine, Beijing University of Chinese Medicine, 100029 Beijing, China

**Keywords:** Cell death, Predictive markers

## Abstract

Intestinal ischemia-reperfusion (I/R) is a multifaceted pathological process, and there is a lack of clear treatment for intestinal I/R injury. During intestinal I/R, oxidative stress and inflammation triggered by cells can trigger a variety of cell death mechanisms, including apoptosis, autophagy, pyroptosis, ferroptosis, and necrosis. These cell death processes can send a danger signal for the body to be damaged and prevent intestinal I/R injury. Therefore, identifying key regulatory molecules or markers of these cell death mechanisms when intestinal I/R injury occurs may provide valuable information for the treatment of intestinal I/R injury. This paper reviews the regulatory molecules and potential markers that may be involved in regulating cell death during intestinal I/R and elaborates on the cell death mechanism of intestinal I/R injury at the molecular level to provide a theoretical basis for discovering new molecules or markers regulating cell death during intestinal I/R injury and provides ideas for drug development for the treatment of intestinal I/R injury.

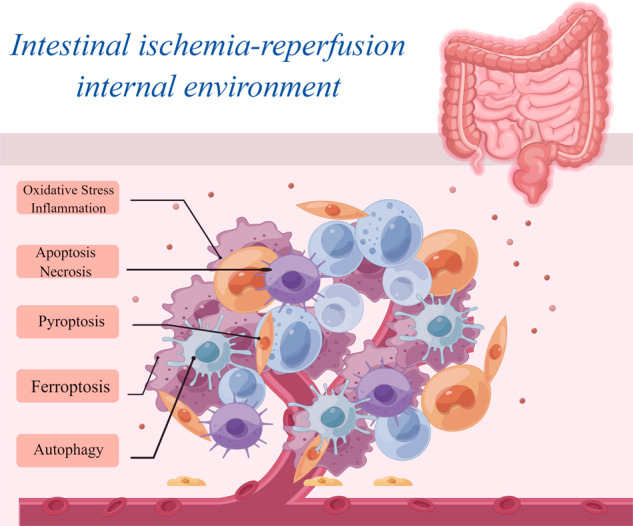

## Facts

- Gaining a deeper understanding of the intricacies surrounding intestinal ischemia-reperfusion injury: elucidating the mechanisms of cell death.
- Emerging therapeutic strategies for mitigating intestinal ischemia-reperfusion injury.
- To explore new therapeutic targets for intestinal ischemia-reperfusion injury from the perspective of oxidative stress and inflammation.

## Open questions

- What is the etiology of intestinal ischemia-reperfusion injury and the efficacy of therapeutic interventions?
- Which additional molecular processes contributing to intestinal ischemia-reperfusion injury remain undisclosed?
- Have reliable biomarkers been identified for intestinal ischemia-reperfusion injury, and are there still challenges present in this particular domain of research?

## Introduction

Intestinal ischemia-reperfusion (I/R) injury is a prevalent acute and critical condition in the clinical setting. It is strongly associated with the onset, progression, and prognosis of various clinical illnesses [1–3]. Intestinal I/R injury is exogenous damage to the intestine that results in fluid extravasation through capillaries and subsequent interstitial edema [4]. Following ischemia and subsequent reperfusion, the permeability of the intestinal capillary increases [5]. Mucosal injury is characterized by the detachment of epithelial cells from the villi, necrosis of epithelial tissue, degradation of the lamina propria, hemorrhaging, and ulceration. These pathological changes together lead to adverse consequences, such as a decrease in nutrient absorption and an increase in the permeability of the mucosal barrier, which facilitates the passage of macromolecules [4, 6]. The activation of self-protective mechanisms in gut cells is a response to and a preventive measure against adverse outcomes [7, 8]. However, a definitive program and standard for the clinical management of intestinal I/R injury are lacking.

When intestinal I/R injury occurs, a cascade of cell death mechanisms is initiated in the body in response to stress. The initiation of these cell death mechanisms is closely related to both the ischemia and reperfusion stages [9, 10]. Intestinal ischemia occurs due to reduced or interrupted blood supply to the intestine, leading to insufficient oxygen and nutrient delivery to cells. This results in the disruption of energy metabolism and impairment of mitochondrial function. Within this ischemic milieu, cellular metabolic activities are compromised, the energy supply is disrupted, and cellular damage occurs [4, 11, 12]. The activation of multiple signaling pathways leads to the initiation of various mechanisms of cell death. Upon reperfusion of blood into the intestine, compromised cells are exposed to an oxygen-enriched milieu, which promotes an inflammatory reaction facilitated by the accumulation of metabolites generated during ischemia and the release of oxygen free radicals [13]. These inflammatory responses can induce cell death by activating factors and signaling pathways linked to different forms of cell death. The mechanisms underlying cell death related to intestinal I/R injury can be categorized primarily into programmed and non-programmed forms. Programmed cell death includes apoptosis, autophagy, pyroptosis, and ferroptosis, whereas non-programmed cell death primarily refers to necrosis.

However, whether programmed cell death or non-programmed cell death occurs, these cell death pathways cannot operate autonomously in the context of intestinal I/R injury [14, 15]. In individuals with intestinal ischemia, affected cells experience inadequate oxygen and nutrient supplies, leading to energy metabolism disturbances and an unstable intracellular milieu. These changes promote programmed cell death [16]. During post-ischemic reperfusion, stimulation and damage to the intestine increase, primarily due to the buildup of metabolites generated during ischemia and the release of oxygen free radicals. Under such conditions, the gut undergoes non-programmed cell death. Although a direct causal connection between these forms of cell death has not been determined, they can also interact [17, 18]. Thus, the intricate interplay between different cell death modalities and their corresponding target genes in the context of intestinal I/R injury needs to be elucidated and summarized.

In this study, we comprehensively analyzed the classical cellular mechanisms associated with intestinal I/R that have been described in recent years. We examined and integrated potential targets associated with these mechanisms. This study might provide a foundation for future research in the field of intestinal I/R injury.

## Oxidative stress and inflammation

Oxidative stress is characterized by an imbalance between oxidation and antioxidant processes in the body. The condition promotes oxidation and the infiltration of neutrophils, an increase in the secretion of proteases, and the generation of numerous oxidative intermediates [19]. The body produces reactive oxygen species (ROS) and reactive nitrogen species (RNS) as a response to detrimental stimuli originating from internal and external environments. [19, 20]

Inflammation is a ubiquitous physiological response that occurs in vascularized living tissues as a defensive mechanism to protect against various injurious stimuli. It occurs in almost all instances of tissue injury in humans, including infections, trauma, diseases, and other stimuli that elicit a bodily reaction. Thus, it is a universally occurring phenomenon [21, 22].

Oxidative stress and inflammation, which affect each other under physiological and pathological conditions and jointly participate in the occurrence and development of many diseases, are closely related. Oxidative stress can cause damage to biomolecules such as cell membranes, DNA, and proteins, thus triggering an inflammatory response. During inflammation, white blood cells release a range of ROS and free radicals to destroy pathogens or remove damaged tissue. However, an excessive inflammatory response can also lead to an increase in oxidative stress. Overall, maintaining the redox balance in the body and a moderate inflammatory response are essential for health [23, 24].

### Intestinal I/R and oxidative stress

During intestinal ischemia, an insufficient blood supply impedes oxygen transport to intestinal cells, which causes a poor oxygen supply to cells and hinders normal oxidative metabolism. Consequently, mitochondria play a key role in REDOX reactions and produce a large number of oxygen free radicals and other ROS under hypoxic conditions [25, 26]. When blood is reintroduced into ischemic intestinal tissue, it replenishes oxygen in the cells. However, the surplus oxygen supplied during this period might induce oxidative stress [27, 28]. Hence, the induction of oxidative stress during intestinal I/R injury may trigger a cascade of signaling pathways and inflammatory reactions, including nuclear factor-κB (NF-κB), NADPH oxidase (NOX), and apoptosis signal-regulated kinase (ASK). After activation, these pathways exacerbate oxidative stress, which results in a detrimental cycle, eventually leading to cell impairment and death.

### Intestinal I/R and inflammation

Intestinal I/R injury-induced inflammation is mainly related to the release of inflammatory mediators, the activation and infiltration of leukocytes, and the release of ROS [29–31]. Intestinal I/R induces the synthesis and release of inflammatory mediators, such as cytokines and chemical mediators [32, 33]. These inflammatory mediators elicit vascular dilation, leukocyte infiltration, platelet activation, and other inflammatory responses, which exacerbate the impairment of the intestinal mucosal barrier and promote the progression of inflammatory reactions [31, 34]. The presence of inflammatory cells, inflammatory mediators, and free radicals can harm the structural integrity of intestinal tissue, which compromises the integrity of the intestinal mucosal barrier and increases mucosal permeability [30, 35]. This process facilitates the infiltration of intestinal bacteria and toxins and subsequently leads to the activation of the immune system and pro-inflammatory cells. These changes eventually lead to inflammatory damage [36, 37].

### Oxidative stress and inflammation in intestinal I/R injury

**Fig. 1:**
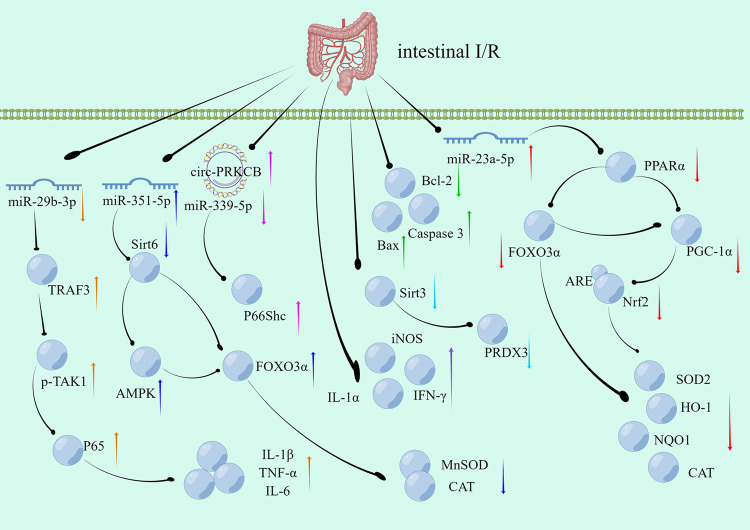
Signaling pathways that may be triggered by cellular oxidation or inflammation during intestinal I/R. The up/down arrows represent an increase/decrease in the expression of the genes during intestinal I/R injury. Arrows of the same color represent the same signaling pathway. I/R ischemia-reperfusion, miRNA microRNA, TRAF3 TNF receptor-associated factor 3, TAK1 TGF-α-activated kinase 1, IL-1β Interleukin-1beta, IL-6 Interleukin-6, Sirt6 Sirtuin-6, AMPK AMP-activated protein kinase, MnSOD manganese-dependent superoxide dismutase, CAT catalase, circRNA circular RNA, PRKCB protein kinase C beta, p66Shc 66 kDa isoform of the adapter molecule ShcA, IL-1α Interleukin-1alpha, IFN-γ IFN-gamma, SIRT3 sirtuin-3, PRDX3 peroxiredoxins3, PPARα peroxisome proliferators-activated receptor alpha, FOXO3α forkhead box O 3α, PGC-1α peroxisome proliferators-activated receptor γ coactivator 1α, Nrf2 Nuclear factor erythroid 2-related factor 2, SOD2 superoxide dismutase 2, NQO1 NAD(P)H: quinone acceptor oxidoreductase 1, HO-1 heme oxygenase-1.

Hydrogen peroxide (H_2_O_2_)-induced mitochondrial oxidative damage and apoptosis are major risk factors for intestinal I/R injury [38]. A study on the underlying mechanism showed that peroxiredoxin 3 (PRDX3) is involved in intestinal I/R injury (Fig. 1). PRDX3 decreased sirtuin-3 (SIRT3) expression during infection and increased acetylation in Caco-2 cells. The inhibition of SIRTs by nicotinamide (NAM) increased the level of acetylated PRDX3 and impaired its antioxidant activity. Additionally, SIRT3 deacetylation at lysine residue K253 increased in mice, which increased SIRT3 dimerization. Transfection of the K253Q plasmid decreased after 12 h of hypoxia, and 4 h of reoxygenation [8]. Thus, oxidative stress regulates ROS production and intestinal I/R injury [39]. CircRNA sponges can reduce hypoxia/reoxygenation (H/R)-induced ROS overproduction by decreasing mitochondrial superoxide anion (O_2_^−^) levels and NADPH oxidase activity and increasing the expression of antioxidant enzymes (Fig. 1). In vitro, H/R was performed in Caco-2 cells after intravenous injection of miR-339-5p agomir or circ-protein kinase C beta (PRKCB) siRNA. The enhanced PRKCB gene-transcribed circRNA circ-PRKCB acted as an endogenous miR-339-5p sponge to regulate the expression of p66Shc and reduce oxidative stress and I/R injury in vivo and in vitro following intestinal infarction. The study’s findings indicated that circ-PRKCB/miR-339-5p/p66Shc is important for understanding oxidative stress related to intestinal I/R [40]. Several studies on intestinal I/R injury have shown that certain monomers derived from traditional Chinese medicine or non-coding RNAs can regulate intestinal I/R injury via mechanisms involving oxidative stress or the inflammatory response. For example, agmatine can mitigate intestinal I/R injury in rats by reducing oxidative stress and inflammatory responses [41]. Similarly, dioscin can attenuate intestinal I/R injury in mice by modulating oxidative stress mediated by miR-351-5p [42] (Fig. 1). In contrast, miR-23a-5p can promote oxidative stress and exacerbate intestinal I/R injury in mice by targeting peroxisome proliferator-activated receptor alpha (PPARα) [43] (Fig. 1). The activation of the nuclear factor erythroid 2-related factor 2 (Nrf2)/heme oxygenase-1 (HO-1)/NAD(P)H: quinone acceptor oxidoreductase 1 (NQO1) signaling pathway regulates the anti-inflammatory, antioxidant, and anti-apoptotic effects induced by sesamin; thus, this pathway protects against and alleviates intestinal I/R injury in rats [44] (Fig. 1). These findings indicate that oxidative stress and inflammation are involved in intestinal I/R injury. These findings will also contribute to the identification of novel therapeutic targets for treating this pathological condition.

## Programmed and non-programmed cell death

**Fig. 2:**
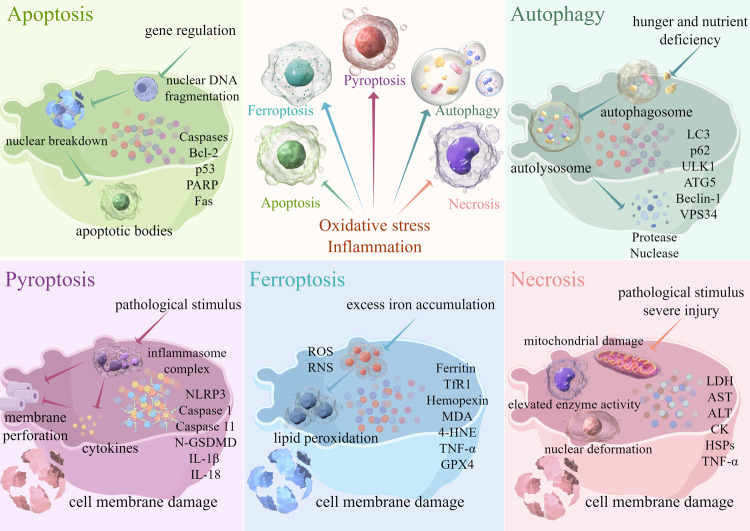
Programmed and non-programmed cell death. The occurrence of programmed (apoptosis, autophagy, pyroptosis, and ferroptosis) and non-programmed (necrosis) cell death elicits various cellular alterations, encompassing the initiation of cell death, modifications in organelles, and alterations in signature proteins. Bcl-2 Bcl-2-associated X protein, p53 Tumor Protein 53, PARP Poly (ADP-Ribose) Polymerase, Fas Fas cell surface death receptor, LC3 Microtubule-associated protein 1 light chain 3, ULK1 Unc-51 Like Autophagy Activating Kinase 1, ATG-5 Autophagy-Related 5, VPS34 Vacuolar Protein Sorting 34, NLRP3 NOD-, LRR- and pyrin domain-containing 3, GSDMD gasdermin D, IL-1β Interleukin-1beta, IL-18 Interleukin-18, TfR1 Transferrin Receptor 1, MDA malondialdehyde, 4-HNE 4-Hydroxynonenal, GPX4 Glutathione Peroxidase 4, ROS Reactive Oxygen Species, LPO malondialdehyde, LDH lactate dehydrogenase, ALT Alanine Aminotransferase, AST Aspartate Aminotransferase, HSPs Heat Shock Proteins, CK creatine kinase, TNF-α tumor necrosis factor-α.

Programmed cell death primarily occurs during the growth and development of certain organisms. Based on certain predetermined requirements, some cells are sacrificed to meet a specific goal [45]. In contrast, in non-programmed cell death, an organism is compelled to sacrifice specific cells due to external factors or certain reasons, often resulting from physiological irregularities [46, 47]. The distinguishing features of programmed and non-programmed cell death are summarized in Fig. 2.

When intestinal I/R injury occurs, a large number of impaired cells undergo apoptosis [48, 49], and the reliance on a single mode of cell death is a serious threat to the intestinal tissue, as inhibiting this pathway can lead to grave consequences. Preserving intestinal integrity requires the action of programmed and non-programmed cell death mechanisms.

### Apoptosis and necrosis

Apoptosis is a regulated form of cell death, which mainly occurs through the activation of the Caspase protease family, leading to the fragmentation of nuclear DNA and eventually cell death. Specifically, apoptosis activates the Caspase protease family through two main pathways (endogenous and exogenous). In the endogenous pathway, mitochondria release cytochrome C and activate Caspase 9. In an exogenous pathway, the death receptor activates Caspase 8. The activated initiating Caspase eventually activates the executive Caspase, resulting in nuclear DNA breakage and ultimately apoptosis [50]. This process plays a key role in maintaining intracellular homeostasis and facilitates intricate operations in multicellular organisms [51]. Apoptosis is an important process that occurs in intestinal tissue. It helps eliminate non-functional, unwanted, abnormal, and detrimental cells. By optimizing the intestinal structure and cell count, apoptosis ensures the appropriate development of the intestine [52, 53].

Necrosis is an uncontrolled, passive process of cell death. It is usually caused by severe cell damage, a process in which cells are irreversibly damaged and eventually die during a pathological process. Necrosis is characterized by swelling of organelles, damage to the plasma membrane, and eventually, cell lysis. Cell contents spill into the surrounding tissue, causing damage to the tissue. Unlike programmed cell death, cell necrosis is primarily caused by harmful agents that invade the body [54, 55].

#### Relationship of intestinal I/R with apoptosis and necrosis

**Fig. 3:**
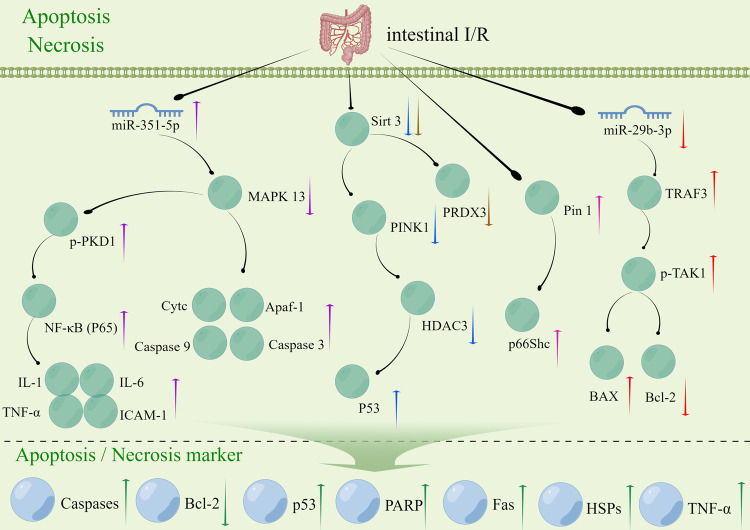
Signaling pathways that may be triggered by cellular apoptosis and necrosis during intestinal I/R. The up/down arrows represent an increase/decrease in the expression of the gene during intestinal I/R injury. Arrows of the same color represent the same signaling pathway. I/R ischemia-reperfusion, miRNA microRNA, AMPK AMP-activated protein kinase, PKD1 polycystic kidney disease 1, ICAM-1 intercellular cell adhesion molecule-1, IL-1 Interleukin-1, IL-6 Interleukin-6, PINK1 Putative kinase 1, HDAC3 Histone deacetylase 3, Pin1 peptidyl-prolyl cis-trans isomerase, p66Shc 66 kDa isoform of the adapter molecule ShcA, PRDX3 peroxiredoxin 3, MAPKA mitogen-activated protein kinase, TRAF3 TNF receptor-associated factor 3, TAK1 TGF-α-activated kinase 1, Bax Bcl-2-associated X protein, Bcl-2 B-cell lymphoma 2, PARP Poly ADP-ribose polymerase, HSPs Heat Shock Proteins.

Ischemia can stimulate the production of deleterious substances and also induce inflammation, apoptosis, and necrosis of epithelial cells [56]. Intestinal I/R injury induces apoptosis and necrosis in the intestinal epithelial cells, which results in impaired intestinal barrier function and ultimately leads to multiple organ dysfunction syndrome [49]. The inflammatory response drives the occurrence of apoptosis and necrosis [57]. Inflammatory mediators and cytokines released during intestinal I/R injury can trigger the activation of signaling pathways associated with apoptosis and necrosis, thereby facilitating the onset of apoptosis and necrosis [58]. The target genes or signaling pathways that can regulate apoptosis and necrosis in intestinal I/R injury include a prolyl-isomerase, peptidyl-prolyl cis-trans isomerase (Pin1), and p66Shc (pink arrow; Fig. 3), SIRT3 and PRDX (the brown arrow, Fig. 3), the SIRT3 and Putative kinase 1 (PINK1)/Histone deacetylase 3 (HDAC3)/p53 (blue arrow; Fig. 3) signaling pathway, the miR-351–5p/mitogen-activated protein kinase (MAPK13) (purple arrow; Fig. 3) signaling pathway, the miR-29b-3p/TNF receptor-associated factor 3 (TRAF3)/TGF-α-activated kinase 1 (TAK1) (red arrow; Fig. 3) signaling pathway, etc. Some drugs, such as Dexmedetomidine and dioscin, can also regulate apoptosis and necrosis associated with intestinal I/R injury.

#### Apoptosis and necrosis in intestinal I/R injury

Intestinal epithelial oxidative stress and apoptosis are the key pathogenic mechanisms underlying intestinal I/R injury. Pin1 was found to regulate the activity of p66Shc during intestinal I/R. This upregulation led to the accumulation of intestinal mitochondrial ROS and the apoptosis of many epithelial cells. Additionally, it increased protein expression and enzyme activity of Pin1, as well as, the interaction between Pin1 and p66Shc. The activation of Pin1 facilitated the translocation of p66Shc to the mitochondria during intestinal I/R, and it also helped to alleviate gut damage and secondary lung injury. These findings suggested that Pin1 inhibition may be a novel prophylactic target for treating intestinal I/R injury [38]. Some traditional Chinese medicine monomers or non-coding RNAs can regulate intestinal I/R injury through apoptosis or necrosis. For example, Dexmedetomidine can protect rats against intestinal I/R injury by promoting mitophagy and inhibiting the apoptosis of enteric glial cells (EGCs) through inhibit the SIRT3/PINK1/HDAC3/p53 pathway [59]. Dioscin can alleviate intestinal I/R injury in mice by regulating miR-351-5p/MAPK13-mediated inflammation and apoptosis [60]. SIRT3-mediated deacetylation of PRDX3 can decrease mitochondrial oxidative damage and apoptosis induced by intestinal I/R injury in mice [8]. The findings of these studies indicated that apoptosis and necrosis can significantly contribute to intestinal I/R injury.

In conclusion, studies on intestinal I/R injury and its association with apoptosis and necrosis have found that during the ischemic phase, cellular damage occurs, followed by inflammatory responses during reperfusion, which leads to tissue necrosis [58, 61]. Inflammation plays a key role in intestinal I/R injury, regardless of whether apoptosis or necrosis is involved. To better understand the mechanisms underlying apoptosis and necrosis in intestinal I/R injury, changes in inflammatory markers need to be monitored. The association between intestinal I/R injury and cell death mechanisms like apoptosis and necrosis needs to be further studied.

### Pyroptosis

Pyroptosis is a mode of programmed death of inflammatory cells that occurs mainly through the activation of various Caspases, including Caspase 1, mediated by inflammatory bodies. Pyroptosis leads to the shear and polyaggregation of various Gasdermin family members, including gasdermin D (GSDMD), resulting in cell perforation and cell death [62]. Pyroptosis is faster than apoptosis and is accompanied by the release of a large number of pro-inflammatory cytokines [63]. When pyroptosis occurs, cells swell. Before the cell ruptures, protrusions form on the cell, and pores form on the cell membrane, which causes the cell membrane to lose its integrity and release its contents, resulting in inflammation. Under such conditions, the nucleus is located in the center of the cell [62, 64].

#### Intestinal I/R and pyroptosis

**Fig. 4:**
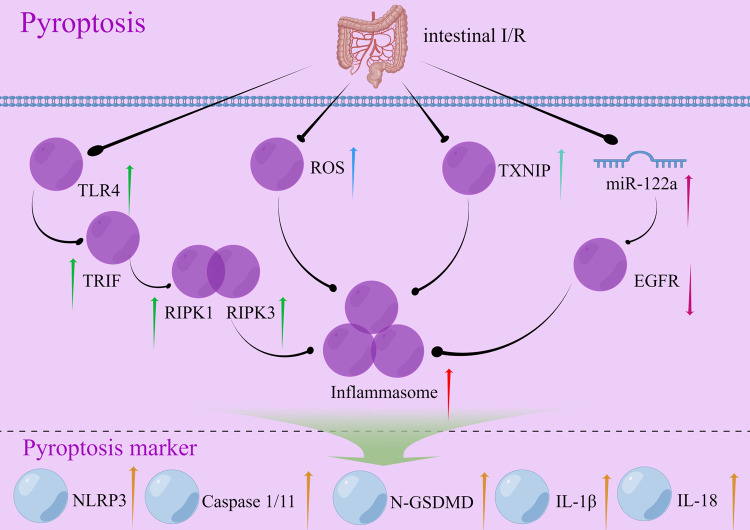
Signaling pathways that may be triggered by cellular pyroptosis during intestinal I/R. The up/down arrows represent an increase/decrease in the expression of the gene during intestinal I/R injury. Arrows of the same color represent the same signaling pathway. I/R ischemia-reperfusion, TLR Toll-like receptor, TRIF Toll/IL-1 receptor (TIR) domain-containing adapter, RIPK receptor-interacting serine/threonine-protein kinase-1, ROS Reactive Oxygen Species, TXNIP Thioredoxin-interacting protein, miRNA microRNA, EGFR epidermal growth factor receptor, NLRP3 NOD-, LRR-, and pyrin domain-containing 3, ASC Apoptosis-associated speck-like protein containing a caspase recruitment domain, GSDM Gasdermin, IL-18 Interleukin-18, IL-1β Interleukin-1beta.

In intestinal I/R, hypoxia, and reperfusion cell damage may cause pyroptosis. Unlike apoptosis, which is a controlled cell death process, pyroptosis is an uncontrolled form of cell death that occurs when the gut is severely damaged by cells. Pyroptosis is a major risk factor for intestinal barrier destruction and cell death [65]. The occurrence of pyroptosis in intestinal I/R injury was confirmed by histopathological findings and intestinal barrier indices, including transepithelial electrical resistance (TER), tight-junction protein, and serum biomarkers. These findings indicated that I/R injury caused intestinal barrier destruction and cell death [65]. Studies on pyroptosis have focused on the classical signaling pathway induced by exogenous injury [62], although both classical and non-classical signaling pathways are present. Understanding the link between intestinal I/R and pyroptosis can help in developing preventive and therapeutic strategies to reduce the adverse effects of intestinal I/R injury in patients. Recent studies on intestinal I/R injury have identified several target genes and signaling pathways involved in the regulation of pyroptosis, including Thioredoxin-interacting protein (TXNIP) (light blue arrow; Fig. 4), miR-122a, and epidermal growth factor receptor (EGFR) (purple arrow; Fig. 4), Toll-like receptor4 (TLR4), Toll/IL-1 receptor domain-containing adapter (TRIF), receptor-interacting serine/threonine-protein kinase-1 (RIPK1), RIPK3 (dark green arrow; Fig. 4), ROS (blue arrow; Fig. 4), etc. The traditional Chinese medicine Corilagin (Cor) can also treat intestinal I/R injury by regulating pyroptosis.

#### Pyroptosis in intestinal I/R injury

The hypoglycemic drug metformin protects the barrier function of intestinal I/R injury by controlling pyroptosis [65]. Additionally, it actively participates in the suppression of pyroptosis-associated proteins, such as NLRP3 (NOD-, LRR- and pyrin domain-containing 3), Cleaved Caspase 1, and the N-terminus of GSDMD. Metformin can suppress the expression of TXNIP and its interaction with NLRP3. The protective effects of metformin disappeared by siRNA knockdown. This implies that the primary mechanism by which metformin exerts its protective effects against intestinal I/R injury is through its interaction with TXNIP [65]. This was the first study in the field of intestinal I/R injury to show that pyroptosis is involved in the process of injury. This finding provided the foundation for subsequent research on the role of pyroptosis in intestinal I/R injury. A study found that increasing the expression of miR-122a significantly inhibits the activity of EGFR in intestinal I/R injury, reduces the expression of EGFR mRNA and protein, and increases the expression of NLRP3 mRNA and protein. The expression of Caspase 1, N-GSDMD, ASC, IL-1β, and IL-18 proteins was upregulated to promote pyroptosis [66]. Thus, miR-122a is essential for regulating intestinal I/R injury; the study also found that miR-122a promotes pyroptosis by inhibiting the EGFR-NLRP3 signaling pathway in mice [66]. Cor is a natural ellagitannin found in various plants and has many biological and pharmacological properties [67]. It can significantly reduce intestinal I/R-induced pathological injury, inflammatory response, oxidative stress, NLRP3 inflammasome activation, and pyroptosis in intestinal and lung tissues both in vivo and in vitro. Thus, Cor may be an effective therapeutic agent for treating intestinal I/R-induced inflammation and tissue injury [68]. These studies focused on investigating the mechanism by which pyroptosis affects intestinal I/R injury and identifying potential therapeutic targets.

The findings of the above-mentioned studies indicated that the assembly and activation of the NLRP3 inflammasome strongly influence the initiation of pyroptosis, irrespective of alterations in the target genes or pathways governing pyroptosis [69, 70]. Hence, alterations in the NLRP3 inflammasome need to be elucidated to identify potential targets for the regulation of pyroptosis. Although the investigation of the effects of pyroptosis on intestinal I/R injury started later than studies on apoptosis and necrosis, it has received increasing attention. Pyroptosis is considered to be an important mechanism in the study of intestinal I/R injury.

### Ferroptosis

Ferroptosis is a regulated form of cell death characterized by a lethal threshold of iron-dependent increase in lipid peroxidation. The inhibition of cystine transport proteins, such as Erastin, leads to the depletion of intracellular Glutathione (GSH), which leads to the inactivation of Glutathione Peroxidase 4 (GPX4) [71, 72]. This inactivation increases lipid peroxidation, which partially induces cell death. The direct inhibition of GPX4, caused by RSL3, also leads to this effect [73, 74]. Several biological processes are highly susceptibility to ferroptosis, including the metabolism of amino acids, iron, and polyunsaturated fatty acids, as well as the biosynthesis of GSH, phospholipids, NADPH, and coenzyme Q10 [73, 75]. Several studies have found a correlation between intestinal I/R injury and ferroptosis, and some studies have also provided evidence that ferroptosis is involved in intestinal I/R injury [74, 76].

#### Intestinal I/R and ferroptosis

**Fig. 5:**
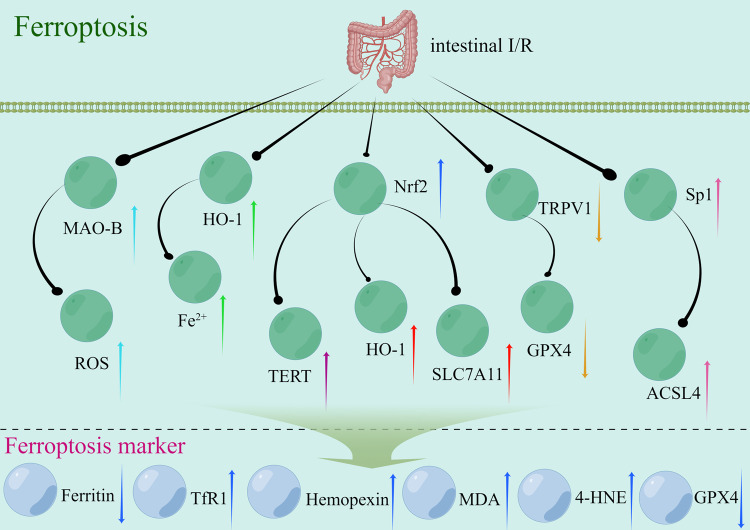
Signaling pathways that may be triggered by cellular ferroptosis during intestinal I/R. The up/down arrows represent an increase/decrease in the expression of this gene during intestinal I/R injury. Arrows of the same color represent the same signaling pathway. I/R ischemia-reperfusion, MAO-B monoamine oxidase b, ROS Reactive Oxygen Species, HO-1 heme oxygenase 1, Nrf2 Nuclear factor erythroid 2-related factor 2, TERT telomerase reverse transcriptase, TRPV1 transient receptor potential cation channel subfamily V member 1, TfR1 Transferrin Receptor 1, MDA Malondialdehyde, 4-HNE 4-Hydroxynonenal, Gpx4 glutathione peroxidase 4, Sp1 special protein 1, ACSL4 Acyl-CoA synthetase long-chain family member 4.

The main factors contributing to ferroptosis in intestinal I/R injury include oxidative stress, the release of iron ions, lipid peroxidation, and iron dependence [76–79]. Intestinal I/R injury induces oxidative stress, characterized by the excessive accumulation of reactive oxygen species generated intracellularly. Excessive ROS production disrupts the cellular REDOX equilibrium [79]. Iron, which serves as a key catalyst and oxidizing agent, participates in the generation of free radicals, thereby increasing oxidative stress [80]. Iron ions are released after cell injury and the subsequent disruption of cell membranes and histiocytosis during intestinal I/R [78]. Iron ions, in an unbound state, can interact with ROS and enhance oxidative stress. Iron-induced oxidative stress increases lipid peroxidation [76, 81]. Under oxidative stress, a large number of ROS can engage in reactions that disrupt the integrity of cellular lipid structures, which damage and impair the functions of the cell membrane [26]. The occurrence of ferroptosis depends on the presence of free iron in the cellular milieu. In the context of intestinal I/R injury, ferroptosis might be a prominent mechanism of cell death caused by the disruption of tissue integrity and the subsequent release of free iron ions [76]. Studies have identified specific genes and signaling pathways, such as special protein 1 (Sp1) (pink arrow; Fig. 5), transient receptor potential cation channel subfamily V member 1 (TRPV1) (brown arrow; Fig. 5), the Nrf2 signaling pathway (dark blue arrow; Fig. 5), HO-1 (green arrow; Fig. 5), the Nrf2/telomerase reverse transcriptase (TERT) signaling pathway (purple arrow, Fig. 5), and monoamine oxidase b (MAO-B) (blue arrow; Fig. 5), which can regulate ferroptosis associated with intestinal I/R injury. Additionally, certain traditional Chinese medicines, such as capsiate (CAT) and Apigenin-7-O-β-D-(-6″-p-coumaroyl)-glucopyranoside (APG), can regulate ferroptosis induced by intestinal I/R injury.

#### Ferroptosis in intestinal I/R injury

Acyl-CoA synthetase long-chain family member 4 (ACSL4) is a key enzyme that regulates lipid composition during reperfusion. Sp1 is a crucial transcription factor that can increase the transcription of ACSL4 by binding to the ACSL4 promoter region. This interaction provides a unique and effective mechanism for preventing intestinal I/R injury by controlling ferroptosis [76]. Some intestinal metabolites or traditional Chinese medicine monomers can also regulate intestinal I/R injury through ferroptosis. For example, some studies investigated the changes in intestinal flora and metabolites in the process of intestinal I/R [3, 82, 83], as well as, the protective effects of CAT. CAT is a natural plant compound related to chili peppers and belongs to the capsaicin family [84]. The results showed that CAT is a metabolite of the intestinal flora, and preoperative fecal CAT level in patients with cardiopulmonary bypass is negatively correlated with intestinal I/R injury. However, the glutathione peroxidase 4 (Gpx4) inhibitor RSL3 is inhibited by CAT. The TRPV1 antagonist JNJ-17203212 can enhance Gpx4 expression and inhibit iron poisoning by activating TRPV1, which provides a potential pathway for intestinal I/R treatment [84]. APG is a new flavonoid glycoside isolated from *Clematis tangutica* that has strong antioxidant abilities [85]. Studies have investigated the effect of APG on intestinal I/R in vivo and in vitro and found that APG can significantly improve intestinal edema and Chiu’s score in mice. It can reduce ROS production and Fe^2+^ accumulation, maintain mitochondrial function, and inhibit ferroptosis [86]. These studies showed that ferroptosis is associated with intestinal I/R injury. Their findings provide a fundamental basis for understanding the role of ferroptosis in this specific pathological condition. They also provide valuable insights into and avenues for future investigations on the involvement of ferroptosis in intestinal I/R injury.

Ferroptosis is a form of iron-dependent cell death [87]. To summarize, intestinal I/R-induced oxidative stress, release of iron ions, lipid peroxidation, dependence on iron, and other factors may contribute to iron death [76, 88, 89]. Recent studies have shown that oxidative stress might be the most plausible determinant of iron death in the context of intestinal I/R injury [79]. When investigating ferroptosis, the effect of oxidative stress needs to be accounted for, and pertinent markers of oxidative stress need to be evaluated. However, ferroptosis, which is a novel form of cell death, has received limited attention in the context of I/R injury. Thus, its role and underlying mechanisms associated with I/R injury need to be comprehensively investigated.

### Autophagy

Autophagy is a vital intracellular degradation process through which damaged or excessive proteins, organelles, and intracellular waste are sequestered within autophagosomes. These autophagosomes subsequently fuse with lysosomes, which leads to the breakdown of their contents into essential molecular components that can be used for cellular recycling [90]. Autophagy helps maintain cellular integrity, facilitate cell viability during nutrient scarcity, and react to cytotoxic agents. Autophagy includes constitutive autophagy, which operates under regular physiological circumstances, and inducible autophagy, which is activated in response to stress [91, 92]. Constitutive autophagy helps in self-preservation by facilitating the growth and development of cells, protecting cells from metabolic stress and oxidative harm, and playing a vital role in maintaining cellular homeostasis. It also helps in the synthesis, degradation, and recycling of cellular constituents. However, excessive autophagy can result in metabolic stress, the degradation of cellular constituents and, ultimately, cell death [90, 92]. Several studies have shown that autophagy participates in various physiological and pathological processes, including cellular equilibrium, aging, immune responses, tumor formation, and intestinal I/R injury [93, 94].

#### Intestinal I/R and autophagy

**Fig. 6:**
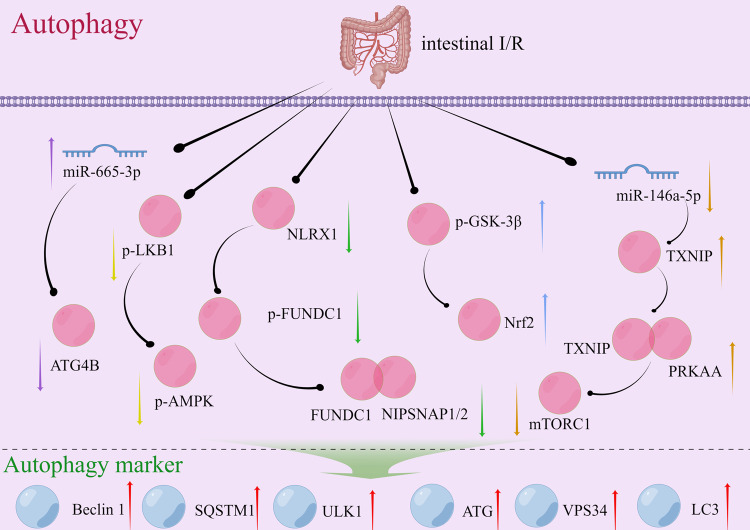
Signaling pathways that may be triggered by cellular autophagy during intestinal I/R. The up/down arrows represent an increase/decrease in the expression of the gene during intestinal I/R injury. Arrows of the same color represent the same signaling pathway. I/R ischemia-reperfusion, miRNA microRNA, ATGs autophagy-related genes, LKB1 Liver Kinase B1, AMPK AMP-activated protein kinase, NLRX1 NOD-like receptor X1, FUNDC1 FUN14 domain-containing 1, NIPSNAP 1 and 2 nitrophenylphosphatase domain and non-neuronal SNAP25-like protein homolog 1 and 2, LC3 II Microtubule-associated protein 1 light chain 3 II, SQSTM1/p62 sequestosome 1, GSK-3β Glycogen synthase kinase 3 beta, Nrf2 Nuclear factor (erythroid-derived 2)-like 2, TXNIP Thioredoxin-interacting protein, PRKAA protein kinase AMP-activated catalytic subunit alpha, mTORC1 mechanistic target of rapamycin kinase complex 1, ULK1 Unc-51 Like Autophagy Activating Kinase 1, VPS34 Vacuolar Protein Sorting 34.

Autophagy may be induced during intestinal I/R to eliminate impaired cellular constituents and maintain cell viability [95]. Intestinal ischemia disrupts energy metabolism and destabilizes the intracellular milieu. Cells may perform autophagy to eliminate dysfunctional or anomalous organelles and proteins, thus obtaining energy and nutrients through the degradation of these cellular components to ensure survival. Upon reperfusion of blood flow into the ischemic intestine, an inflammatory response and cellular stress might occur, which might trigger autophagy as a cellular stress response mechanism to regulate the intracellular environment and facilitate cellular adaptation [95, 96]. While examining intestinal I/R injury, autophagy flux was found to be impaired throughout the intestinal I/R procedure [97]. Mitochondrial autophagy plays a key role in the pathogenesis of intestinal I/R injury [98, 99]. Some studies have provided evidence for the existence of target genes or signaling pathways, such as the miR-665–3p/ATG4B (autophagy-related cysteine protein) signaling pathway (purple arrow; Fig. 6), the NOD-like receptor X1 (NLRX1)/FUN14 domain-containing 1 (FUNDC1)**/**nitrophenylphosphatase domain and non-neuronal SNAP25-like protein homolog 1 and 2 (NIPSNAP1/2) signaling pathway (green arrow; Fig. 6), the miR-146a-5p/TXNIP/protein kinase AMP-activated catalytic subunit alpha (PRKAA)**/**mechanistic target of rapamycin kinase (mTOR) signaling pathway (brown arrow; Fig. 6), and the Glycogen synthase kinase 3 beta (GSK-3β)/Nrf2 signaling pathway (blue arrow; Fig. 6). These pathways can regulate autophagy associated with intestinal I/R injury. Additionally, certain traditional Chinese medicines, including paeoniflorin, can modulate autophagy during intestinal I/R injury [95].

#### Autophagy in intestinal I/R injury

Autophagy is predominantly used along with traditional Chinese medicine monomers or non-coding RNA for regulating diseases in studies on intestinal I/R injury. Paeoniflorin, a monoterpene glucoside, has various health benefits, including regulation of autophagy and anti-inflammatory, anti-apoptotic, and antioxidant effects [100, 101]. Paeoniflorin preconditioning can protect rats against intestinal I/R injury by alleviating intestinal histological damage, inflammatory response, oxidative stress, and cell apoptosis, without affecting control cells. Impairments in autophagy can be restored by upregulating the degradation of autophagy-related proteins p62/SQSTM1, the expression of LC3-II and beclin-1, and the synthesis of autophagosome. Some [95] researchers have shown that miRNAs negatively regulate autophagy by targeting ATGs [102]. Autophagy flux can be compromised during intestinal I/R. Additionally, miR-665–3p can negatively affect the expression of ATG4B in Caco-2 and IEC-6 cells, which was shown using ileum biopsy samples obtained from patients diagnosed with intestinal infarction. In vivo, locked nucleic acid-modified inhibition reduces I/R-induced systemic inflammation and apoptosis by restoring the autophagy flux [97]. In the mitochondria, NLRX1 is highly expressed in the gut and can regulate ROS production, mitochondrial damage, autophagy, and apoptosis [103, 104]. Some studies examined the role of NLRX1 in mitochondrial homeostasis and apoptosis after intestinal I/R injury and found that NLRX1 was significantly downregulated after intestinal I/R injury [98]. The miR-146a-5p/TXNIP axis can also reduce intestinal I/R injury in mice by inhibiting autophagy through the PRKAA/mTOR signaling pathway [94]. Ischemic postconditioning (IPO) is a treatment method for I/R injury. The core of IPO is to mitigate reperfusion injury through short, alternating blood flow blockade and restoration during the reperfusion stage [105]. Research has shown that IPO can effectively protect intestinal I/R injury from ischemic postconditioning by inducing autophagy, activating Akt, inactivating GSK-3β, and activating Nrf2. These studies found that autophagy strongly influences the development and progression of intestinal I/R injury, serving as a well-established cellular protective mechanism.

To summarize, stimulating autophagy during intestinal I/R allows cells to cope with oxidative stress and other forms of damage, which reduces the likelihood of cell death [65, 95]. The activation of autophagy in response to intestinal I/R serves as a cell survival mechanism. It facilitates the elimination of impaired cellular constituents and cellular adaptation [65, 106]. A comprehensive understanding of the association between intestinal I/R and autophagy provides insights into the underlying pathophysiological mechanisms and novel strategies for designing therapeutic interventions.

## Discussion

Intestinal I/R is the restoration of blood flow to the intestine following a period of inadequate blood supply, which can occur in various clinical scenarios, such as intestinal obstruction, thrombosis, and insufficient arterial blood supply. The resultant tissue hypoxia and nutrient insufficiency stemming from intestinal ischemia can lead to several pathophysiological mechanisms, including disturbances in cellular energy metabolism, impairment of mitochondrial function, and generation of oxygen free radicals, among others [2, 77]. These alterations increase intracellular stress, subsequently resulting in cell death. Upon reperfusion, the ischemic region is replenished with blood, which allows cells to regain access to oxygen and nutrients. However, this process might be harmful as the reintroduction of blood to ischemic tissue during reperfusion may lead to reperfusion injury, and eventually, cell death [4, 107].

Several studies have shown that intestinal I/R injury induces cell death via two primary pathways, including oxidative stress and inflammatory response [65, 107–111].

Upon reperfusion, oxygenated environments supply tissues with high levels of oxygen, which promotes the generation of oxygen free radicals. These radicals, characterized by their high reactivity, engage with intracellular biological macromolecules, including proteins, lipids, and nucleic acids, thus increasing oxidation reactions that lead to cell impairment and death. Oxidative stress negatively affects various cellular components, such as membrane lipids, mitochondria, and DNA, thus compromising their structural integrity and hindering their functions. The cumulative effect of these direct and indirect consequences leads to cell death [65, 107, 110, 111]. Reperfusion following ischemia can elicit an inflammatory response. After the reintroduction of blood flow to the ischemic region, immune cells, i.e., neutrophils and macrophages, become activated and release inflammatory mediators, which include inflammatory cytokines and chemokines. The excessive release of these inflammatory mediators increases inflammatory reactions, which promotes the infiltration of inflammatory cells and elicits a tissue inflammatory response, eventually leading to cell death [108, 109].

Intestinal I/R injury is closely associated with various modes of cell death, where oxidative stress and inflammation serve as the underlying mechanisms following abnormal changes in the intestinal environment [40, 49, 109, 111, 112].

Several studies have found a strong correlation between intestinal I/R and programmed cell death, specifically apoptosis. Intestinal ischemia disrupts the oxygen and nutrient supply to tissues, which compromises cell survival and results in pathophysiological alterations, including energy metabolism disruption, mitochondrial dysfunction, and an increase in intracellular stress. These factors collectively activate the apoptosis signaling pathway, which induces cells to enter the apoptotic state. When blood flows back into the ischemic intestinal tissue during reperfusion, the apoptotic signaling pathway might be further activated, thus accelerating apoptosis. During reperfusion, oxidative stress and inflammation can influence apoptotic signaling pathways in various ways, stimulating cells to enter an apoptotic state [49, 109, 112].

Several studies have found a correlation between intestinal I/R and non-programmed cell death. [113, 114] In contrast to programmed cell death, such as apoptosis, non-programmed cell death does not follow a pre-established intracellular signaling cascade. Necrosis is a common form of non-programmed cell death. During intestinal I/R, cells might get damaged due to ischemia, which can lead to non-programmed cell death during the subsequent reperfusion phase. Reperfusion injury manifests as necrosis, characterized by non-programmed cell death. The incidence of reperfusion injury is closely associated with various factors, such as oxidative stress, inflammation, and mitochondrial dysfunction. The detrimental effects of oxidative stress-induced oxygen free radicals and inflammatory response-released inflammatory mediators affect the cell membrane, organelles, and key cellular molecules, which lead to non-programmed cell death [40, 111, 112].

To summarize, the occurrence of intestinal I/R can elicit cell death via oxidative stress and inflammatory response. Oxygen free radicals induce oxidative stress, and along with inflammatory responses elicited by inflammatory mediators, they exert direct and indirect harmful effects on cell structure and functionality, ultimately leading to cell death. Although we tried to clarify the mechanism of cell death induced by oxidative stress and inflammation during intestinal I/R injury, we did not thoroughly analyze the specific induction, mechanism or potential target of cell death during intestinal I/R injury, and the cause of cell death has not been fully elucidated. In future studies, more comprehensive studies on the triggers, mechanisms and targets of cell death during intestinal I/R generation are needed, as these studies may help elucidate the complex mechanisms of intestinal I/R injury. Also, more targets need to be identified, and better therapeutic interventions need to be developed to address the unanswered questions in this field of research.
